# The Integrated Hemostatic Network in Pediatric ECMO: A Systems-Level Framework for Precision Hemostasis

**DOI:** 10.3390/children13101441

**Published:** 2026-10-21

**Authors:** Lucio F. P. de Lima, Isabella A. Martins, Denise V. Louzada, Patricia T. K. Yonamine

**Affiliations:** 1 Pediatric Intensive Care Medicine (PICU), Hospital SEPACO, São Paulo 04102-002, SP, Brazil; dvlouzada@sepaco.org.br (D.V.L.); ptkyonamine@sepaco.org.br (P.T.K.Y.); 2 Pediatric Hematology, Hospital SEPACO, São Paulo 04102-002, SP, Brazil; iamartins@sepaco.org.br

**Keywords:** pediatric ECMO, Integrated Hemostatic Network, dyshemostasis, hemostasis, anticoagulation, thromboinflammation, bleeding, thrombosis, precision hemostasis

## Abstract

**Highlights:**

**What are the main findings?**

- Pediatric ECMO-associated dyshemostasis is a dynamic network disorder arising from interactions among developmental hemostasis, endothelial dysfunction, coagulation, fibrinolysis, inflammation, and circuit-related perturbations.
- Bleeding, thrombosis, and mixed manifestations reflect evolving clinical states of Integrated Hemostatic Network dysfunction, shaped by patient-specific factors and circuit-related processes rather than anticoagulation intensity alone.

**What are the implications of the main findings?**

- The Integrated Hemostatic Network provides a unified conceptual framework for interpreting ECMO-associated dyshemostasis beyond isolated laboratory abnormalities and anticoagulation targets.
- Integrating clinical assessment, conventional and functional hemostatic testing, developmental context, and circuit evaluation may support individualized, mechanism-directed management and serial reassessment.

**Abstract:**

Pediatric extracorporeal membrane oxygenation (ECMO) is associated with complex hemostatic disturbances arising from interactions among blood–circuit interfaces, developmental hemostasis, endothelial dysfunction, coagulation, fibrinolysis, inflammation, and disease- and treatment-related factors. Conventional approaches focused on individual laboratory abnormalities may not adequately capture this complexity. This review proposes the Integrated Hemostatic Network (IHN) as a unified conceptual framework for understanding ECMO-associated dyshemostasis and informing precision hemostatic management. This narrative review synthesized literature identified iteratively through PubMed searches addressing pediatric ECMO hemostasis and related mechanistic and clinical domains. Priority was given to pediatric studies, prospective comparative studies, randomized trials, systematic reviews, and consensus or guideline documents. Adult and non-ECMO evidence was considered when relevant pediatric data were limited. The evidence was integrated across developmental hemostasis, blood–circuit interactions, platelet and von Willebrand factor biology, coagulation and anticoagulant pathways, fibrinolysis, endothelial dysfunction, inflammation, complement activation, and hemolysis, with implications for multimodal monitoring and individualized management. The evidence supports a systems-level interpretation of pediatric ECMO-associated dyshemostasis, in which interacting determinants evolve over time. Bleeding, thrombosis, and their coexistence may arise from overlapping disturbances rather than isolated abnormalities or anticoagulation intensity alone. Three proposed working hemostatic states—bleeding-predominant, thrombosis-predominant, and mixed bleeding–thrombotic—provide a conceptual structure for clinical assessment and therapeutic reasoning but are not validated biological categories, prediction models, or treatment algorithms. The IHN integrates developmental and clinical context, laboratory and functional assessment, anticoagulant effects, and patient and circuit findings to support longitudinal interpretation beyond isolated laboratory targets. By explicitly integrating these interacting determinants and their temporal evolution, the IHN provides a unified conceptual architecture for understanding pediatric ECMO-associated dyshemostasis. Its clinical utility requires prospective multicenter evaluation to establish reproducibility and determine whether its application improves patient-centered outcomes.

## 1. Introduction

Extracorporeal membrane oxygenation (ECMO) provides life support for neonates and children with refractory respiratory or circulatory failure but remains associated with substantial morbidity and mortality, including neurological injury [1,2,3,4]. Blood–circuit interactions and mechanical forces activate coagulation, platelets, complement, leukocytes, and endothelial pathways, contributing to the hemostatic and inflammatory disturbances associated with ECMO [5,6].

ECMO-associated dyshemostasis cannot be adequately described as a uniform “ECMO coagulopathy.” Developmental factors, underlying disease, systemic inflammation, hemolysis, anticoagulation, and circuit characteristics interact dynamically, producing bleeding, thrombosis, or both [7,8,9,10]. Although established biological models and pediatric ECMO guidance support a multifactorial understanding and multimodal assessment, they do not provide an explicit systems-level architecture linking these determinants to the evolving clinical expression of dyshemostasis [11,12,13].

This review proposes the Integrated Hemostatic Network (IHN), a conceptual framework that explicitly integrates developmental, biological, laboratory, functional, clinical, and circuit determinants and their temporal interactions. Its novelty lies not in recognizing the multifactorial nature of dyshemostasis or the value of multimodal monitoring, but in organizing these interconnected elements into a unified and potentially testable framework. The IHN encompasses platelet–von Willebrand factor–endothelial biology, coagulation and endogenous anticoagulant mechanisms, fibrinolysis, blood–circuit interactions, mechanical forces, hemolysis, complement, innate immunity, and inflammation. Developmental stage, underlying disease, procedures, ECMO configuration, and therapies may modify their relative contributions over time.

The framework proposes three working hemostatic states—bleeding-predominant, thrombosis-predominant, and mixed bleeding–thrombotic—to organize the clinical assessment of dyshemostasis. These remain unvalidated constructs rather than established biological categories, prediction models, or treatment algorithms. Their reproducibility, biological basis, relationship to outcomes, and clinical utility require investigation. This review examines the biological basis of the IHN, ECMO-associated disturbances, their clinical manifestations, and the potential integration of multimodal assessment to inform individualized hemostatic management.

## 2. Literature Identification and Evidence Synthesis

This article is a narrative review developed to provide a conceptual synthesis of dyshemostasis during pediatric ECMO and to propose the Integrated Hemostatic Network (IHN) framework. The literature was identified iteratively through PubMed searches addressing the principal domains of the review, including pediatric ECMO hemostasis, developmental hemostasis, coagulation, platelet and von Willebrand factor biology, endothelial dysfunction, thromboinflammation, fibrinolysis, hemolysis, anticoagulation monitoring, transfusion, and circuit-related hemostatic disturbances. Initial references included the literature underlying the institutional pediatric ECMO hemostasis protocol and were supplemented by targeted searches addressing specific mechanistic and clinical questions that emerged during development of the review. Reference lists of relevant publications were also examined to identify additional pertinent studies.

Priority was given to pediatric ECMO studies and, when available, prospective comparative studies, randomized trials, systematic reviews, and consensus or guideline documents. Adult ECMO and non-ECMO studies were included when they provided relevant mechanistic or methodological evidence; their indirect applicability to pediatric ECMO was considered during synthesis. Because this was a narrative rather than systematic review, no exhaustive study-level search or formal risk-of-bias assessment was performed. Evidence was interpreted according to study design, population, biological or clinical context, and relevance to pediatric ECMO, with attention to distinguishing established findings from observational associations, mechanistic evidence, and expert consensus. Conceptual elements of the IHN framework were identified separately from directly supported clinical evidence.

## 3. Physiology and Ontogeny of the Integrated Hemostatic Network

Hemostasis is a dynamic, spatially organized process that preserves blood fluidity while enabling localized clot formation, stabilization, and resolution. It depends on coordinated interactions among the endothelium and von Willebrand factor (vWF), platelets, coagulation and endogenous anticoagulant systems, and fibrinolysis [14,15,16,17]. Rather than functioning as isolated pathways, these components operate as an integrated biological system regulated by blood flow, cellular interactions, and inflammatory signaling.

The endothelium regulates this equilibrium by limiting platelet activation and coagulation under physiological conditions while supporting appropriate fibrinolysis. Vascular injury disrupts this balance, enabling vWF-mediated platelet adhesion and tissue factor-dependent coagulation, while activated platelets provide a surface for coagulation reactions and thrombin links platelet activation, coagulation, and fibrin formation [18,19,20,21,22]. Coagulation is also functionally coupled to inflammation and innate immunity, with tissue injury, infection, and exposure to artificial surfaces capable of activating endothelial, platelet, coagulation, complement, and inflammatory pathways [23,24]. The IHN therefore encompasses vascular, hemostatic, inflammatory, and immune processes that collectively influence hemostatic balance.

### Ontogeny as a Modifier of the Integrated Network

The components of the IHN undergo substantial developmental changes from fetal life through childhood and adolescence. Developmental hemostasis reflects age-dependent differences in procoagulant and anticoagulant proteins, platelet biology, endothelial pathways, and fibrinolysis and represents a developmentally regulated equilibrium rather than simply an immature form of adult hemostasis [25,26,27,28].

Developmental stage may modify multiple components of the IHN simultaneously. Neonates and young infants differ from older children in coagulation-factor concentrations, endogenous anticoagulant pathways, platelet biology, and fibrinolysis [25,26,27,28]. In parallel, the impact of ECMO exposure is influenced by body size, circuit surface area, priming volume, and the ratio between extracorporeal and circulating blood volume [29]. Critical illness, inflammation, infection, surgery, trauma, transfusion, and organ dysfunction may further perturb this developmentally regulated system [29].

The principal developmental modifiers and their potential relevance to pediatric ECMO are summarized in Table 1.

## 4. Pathobiology of ECMO-Associated Dyshemostasis

ECMO-associated dyshemostasis reflects the dynamic interaction between extracorporeal exposure and the patient’s underlying biological state. Rather than producing a uniform “ECMO coagulopathy,” ECMO perturbs multiple interconnected components of the IHN, with the magnitude and direction of these effects changing over time. These effects may act in opposing directions, allowing prothrombotic stimuli to coexist with impaired effective hemostasis.

### 4.1. Blood–Circuit Interaction, Mechanical Stress, and Thromboinflammation

The extracorporeal circuit continuously exposes blood to non-endothelialized surfaces and altered flow conditions. Adsorption of plasma proteins to artificial materials creates a biologically active blood–surface interface involving platelet, coagulation, complement, and inflammatory pathways, while pumps, cannulae, oxygenators, circuit geometry, and flow conditions further modify blood and cellular behavior [30,31].

Altered shear is particularly relevant to the vWF–platelet axis. Excessive or sustained shear may promote loss of high-molecular-weight vWF multimers and contribute to acquired von Willebrand syndrome, impairing primary hemostasis [32,33]. Mechanical exposure may also activate platelets while altering receptor and signaling function and contributing to platelet consumption and dysfunction [34,35]. Thus, platelet activation and impaired platelet-mediated hemostasis may coexist during ECMO.

These circuit-derived perturbations occur within a host environment already shaped by critical illness. Inflammation, hypoxemia, ischemia–reperfusion injury, infection, tissue injury, and the underlying endothelial state may modify the response to extracorporeal exposure, while interactions among endothelial, platelet, coagulation, complement, and inflammatory pathways may sustain thromboinflammatory activity [23,24].

### 4.2. Hemolysis, Fibrinolysis, and Hemostatic Reserve

Mechanical erythrocyte injury releases cell-free hemoglobin, heme, and other products that may alter endothelial, platelet, vascular, and inflammatory biology [36,37]. Hemolysis may therefore represent both a marker of mechanical blood trauma and a potential contributor to the dyshemostatic environment.

Fibrinolytic regulation is influenced by coagulation activation, endothelial and inflammatory signaling, and the underlying disease state [38]. Alterations in fibrinolytic and related markers have been described in pediatric ECMO, while prospective adult data suggest an association between fibrinolytic abnormalities and bleeding complications [39,40]. These findings support the concept that fibrinolysis may shift toward either excessive fibrin breakdown or impaired fibrin clearance during ECMO, with the balance changing over time.

Continuous circuit exposure, bleeding, platelet activation and consumption, and coagulation-factor depletion may progressively reduce hemostatic reserve [41,42]. Hemostatic reserve refers to the remaining quantitative and functional capacity to maintain effective hemostasis under ongoing biological and mechanical stress.

### 4.3. Anticoagulation Within the Dyshemostatic Network

Systemic anticoagulation is necessary because the extracorporeal circuit provides a persistent nonphysiological surface that promotes coagulation activation and thrombosis. However, anticoagulation primarily modulates the coagulation cascade and cannot correct disturbances involving platelet function, fibrinogen availability, endothelial integrity, fibrinolysis, or inflammation [43,44]. Thus, thrombosis may develop despite apparently adequate anticoagulation, whereas clinically significant bleeding may occur without excessive anticoagulant effect [45].

### 4.4. Dynamic Network Perturbation

The magnitude and direction of ECMO-associated dyshemostasis are influenced by developmental stage, ECMO configuration, underlying disease, preceding cardiopulmonary bypass, cannulation approach, circuit characteristics, circuit-to-patient size relationship, duration of support, inflammatory and endothelial states, mechanical forces, organ dysfunction, and therapeutic interventions. These factors modify network behavior, and their relative contribution may change throughout ECMO support.

Consequently, bleeding and thrombosis may occur separately, sequentially, or simultaneously, involving the patient, the circuit, or both. The clinical expression of dyshemostasis may evolve as the relative contribution of individual network components and modifiers changes over time.

## 5. Proposed Clinical Hemostatic States in ECMO-Associated Dyshemostasis

ECMO-associated dyshemostasis may be clinically expressed through three proposed working hemostatic states: bleeding-predominant, thrombosis-predominant, and mixed bleeding–thrombotic. These states provide a pragmatic framework for integrating clinical manifestations, laboratory and functional hemostatic findings, and patient- and circuit-related factors to characterize the prevailing clinical expression of dyshemostasis. By contextualizing these findings within the Integrated Hemostatic Network, the framework supports interpretation of the underlying hemostatic disturbances, assessment of their clinical implications, and longitudinal reassessment to inform surveillance, preventive measures, and individualized management. The states are dynamic and context-dependent, and patients may transition between them as their clinical condition and hemostatic balance evolve. They represent conceptual clinical constructs rather than validated biological categories or predictive tools.

### 5.1. Thrombosis-Predominant Hemostatic State

The thrombosis-predominant state describes situations in which thrombogenicity or established thrombosis is the principal current clinical concern. It may arise from patient- or circuit-related prothrombotic factors, coagulation activation, inadequate anticoagulant effect, low-flow or stasis conditions, progressive circuit or oxygenator thrombus formation, patient thrombosis, or an evolving thrombotic trajectory [10,45].

Potential contributing mechanisms include persistent thrombin generation, platelet activation, endothelial disturbance, impaired endogenous anticoagulant mechanisms, altered fibrinolysis, complement activation, inflammation, hemolysis, and circuit-related blood trauma.

Thrombosis should not automatically be interpreted as evidence of inadequate anticoagulation. Patient- and circuit-related contributors should be assessed before increasing anticoagulant intensity, particularly when a reversible source of thrombogenicity can be identified.

### 5.2. Bleeding-Predominant Hemostatic State

The bleeding-predominant state describes situations in which bleeding susceptibility or established bleeding is the principal current clinical concern. It may be recognized before overt bleeding when clinical characteristics, acquired risk factors, objective hemostatic abnormalities, or trajectory indicate substantial bleeding vulnerability [10,45].

Potential contributing mechanisms include thrombocytopenia or platelet dysfunction, acquired von Willebrand abnormalities, hypofibrinogenemia, coagulation-factor depletion, dysregulated fibrinolysis, endothelial injury, hemodilution, and anticoagulant effect. When bleeding occurs, these mechanisms should be considered together with its severity, trajectory, source, and relationship to procedures or ECMO rather than attributing bleeding automatically to excessive anticoagulation.

Neonatal or preterm status, recent cardiopulmonary bypass or major surgery, postcardiotomy ECMO, and anticipated high-risk invasive procedures may substantially increase bleeding vulnerability [25,26,27,28,29,45,46]. These contexts may therefore influence clinical assessment even before overt bleeding or a single diagnostic laboratory abnormality becomes apparent.

### 5.3. Mixed Bleeding–Thrombotic Hemostatic State

The mixed bleeding–thrombotic state describes situations in which bleeding vulnerability or bleeding and thrombogenicity or thrombosis are simultaneously relevant to clinical management. It may present as concurrent manifestations, such as active bleeding with progressive circuit thrombosis, or as objective evidence of processes favoring both bleeding and thrombosis before overt complications develop [10,45].

This state reflects the coexistence of mechanisms that impair effective hemostasis and those that promote thrombosis, including platelet dysfunction or activation, platelet consumption, endothelial injury, coagulation activation, depletion or dysfunction of coagulation factors and fibrinogen, altered fibrinolysis, inflammation, hemolysis, and circuit-related blood trauma.

The mixed state should not be interpreted simply as a rationale for selecting an intermediate anticoagulation target. Instead, the mechanisms and trajectories contributing to both processes should be assessed in parallel, with attention to reversible contributors and avoidance of additional bleeding, thrombotic, or circuit-related harm.

**Figure 1:**
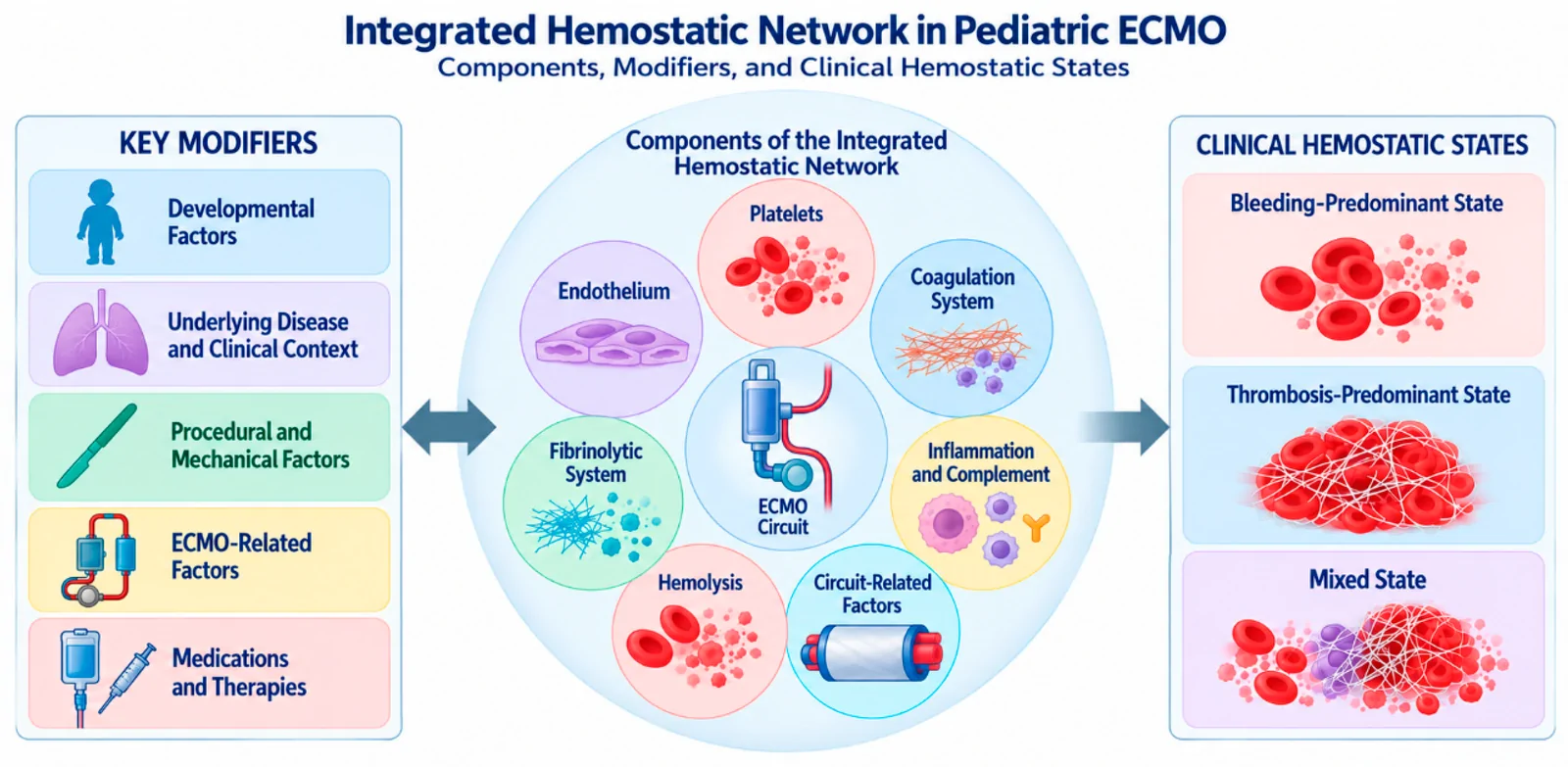
Integrated Hemostatic Network in Pediatric ECMO: components, modifiers, and clinical hemostatic states. The network integrates platelet, endothelial, coagulation, fibrinolytic, inflammatory/complement, hemolytic, and circuit-related components. Key modifiers include developmental factors, underlying disease and clinical context, procedural and mechanical factors, ECMO-related factors, and medications and therapies. The resulting clinical expression is represented as bleeding-predominant, thrombosis-predominant, or mixed bleeding–thrombotic states.

Figure 1 provides a visual synthesis of the Integrated Hemostatic Network framework and its relationship to the prevailing clinical hemostatic state.

### 5.4. From Hemostatic-State Recognition to Integrated Assessment

Hemostatic risk, dyshemostatic processes, and clinical hemostatic states are related but distinct concepts. Hemostatic risk may be substantial before an overt bleeding or thrombotic complication develops, whereas dyshemostatic processes and their clinical expression may evolve over time in response to changes in circuit condition, inflammation, hemolysis, surgery, transfusion, organ function, anticoagulation, and recovery from the underlying disease [45].

Characterization of the prevailing hemostatic state provides a framework for organizing clinical assessment and determining monitoring needs. Laboratory, viscoelastic, clinical, and circuit findings should therefore be interpreted together to characterize the prevailing state and its potential mechanisms [12,13,45]. Because the state is dynamic, reassessment is required when clinically important changes occur.

## 6. Precision Monitoring of Dyshemostasis During Pediatric ECMO

Precision monitoring during pediatric ECMO requires integration of developmental and clinical context, patient- and circuit-related findings, anticoagulant effect, functional hemostatic measurements, and the trajectory of bleeding or thrombosis. The objective is not simply to obtain multiple laboratory measurements, but to characterize the prevailing hemostatic state, identify potentially relevant mechanisms, and recognize clinically meaningful changes over time. The interpretation of individual measurements therefore depends on the context in which they are obtained.

### 6.1. Clinical and Developmental Context

Neonates and preterm infants have a developmentally distinct hemostatic system, while their smaller circulating blood volume and the relationship between circuit volume and patient blood volume may further influence the biological and clinical effects of ECMO exposure [25,26,27,28,29]. Recent cardiopulmonary bypass is also associated with reductions in platelet count, fibrinogen concentration, and related functional measures in children and represents an important bleeding-risk context for postcardiotomy ECMO [46]. Current pediatric consensus similarly emphasizes assessment of coagulation status before major or high-risk procedures during ECMO [13,47].

These factors should therefore be considered modifiers of monitoring interpretation, rather than separate laboratory abnormalities. The same result may have different clinical significance in a stable older child, a neonate, a patient immediately after CPB, or a patient undergoing a high-risk procedure.

### 6.2. Laboratory and Functional Assessment

The principal modalities used during pediatric ECMO are summarized in Table 2. Their roles are complementary: anticoagulation assays primarily estimate drug effect, whereas VET, platelet and fibrinogen measurements, and clinical and circuit assessment provide additional information about hemostatic competence and the consequences of dyshemostasis.

#### 6.2.1. Conventional Anticoagulation Assays

ACT provides rapid whole-blood information on clot initiation but is not specific for UFH and is influenced by platelet count and function, fibrinogen, coagulation factors, temperature, hemodilution, anemia, inflammation, and assay characteristics [12,48,49]. It should therefore be interpreted as a measure of clot initiation rather than a direct measure of heparin effect.

aPTT is widely used to monitor UFH and is also frequently used for bivalirudin. However, its relationship with anticoagulant activity is not fixed and depends on reagent and platform characteristics, calibration, and the patient’s underlying hemostatic status [12,43,50]. Coagulation-factor deficiencies or consumption, inflammation, liver dysfunction, hemodilution, inhibitors, and developmental hemostasis may alter aPTT independently of anticoagulant exposure or modify its response to treatment. Consequently, aPTT should be interpreted using assay-specific institutional targets and in conjunction with clinical findings and complementary measures of anticoagulant effect and hemostatic competence.

Anti-Xa provides a relatively direct estimate of UFH activity through inhibition of factor Xa by the UFH–antithrombin complex. Its interpretation depends on antithrombin availability and assay methodology [51,52]. Assay configurations also differ in whether antithrombin is supplemented exogenously, which can influence measured anti-Xa activity and complicate comparisons across platforms [43,51]. Chromogenic measurements may be affected by hemolysis, hyperbilirubinemia, hypertriglyceridemia, and other preanalytical or platform-specific factors [12,53]. Anti-Xa reflects UFH activity rather than overall hemostatic competence.

Heparin concentration provides an estimate of circulating heparin exposure but does not necessarily reflect biological anticoagulant effect, and methods are not directly interchangeable [54]. It may therefore provide complementary information when conventional anticoagulation assays are discordant.

#### 6.2.2. Viscoelastic Testing

Viscoelastic testing (VET), including TEG and ROTEM, provides a dynamic whole-blood assessment of clot initiation, propagation, strength, stability, and lysis. It incorporates contributions from fibrin and platelets and can therefore complement plasma-based coagulation assays. Pediatric ECMO evidence remains limited and heterogeneous, with interpretation influenced by age, clinical condition, ECMO configuration, platform, reagents, sampling and preanalytical conditions [55,56,57,58,59]. Reproducibility is also an important limitation, and universal pediatric ECMO thresholds or VET-guided anticoagulation algorithms have not been established.

FIBTEM or functional fibrinogen assays may help characterize the contribution of fibrin to reduced clot firmness, while heparinase-modified assays may help assess the contribution of heparin to delayed clot initiation. VET findings should nevertheless be interpreted as complementary functional information rather than as automatic indications for anticoagulant adjustment [12,43,59].

The principal value of VET within the IHN is therefore its ability to identify functional patterns that may help explain abnormalities detected by conventional assays—for example, distinguishing impaired clot strength from isolated prolongation of clot initiation—rather than providing a standalone therapeutic target.

#### 6.2.3. Platelet, Fibrinogen, and PT/INR Assessment

Platelet count provides quantitative information but does not characterize platelet function. Thrombocytopenia may coexist with qualitative dysfunction related to circuit-associated shear and blood–surface interactions [34,35]. Functional methods such as aggregometry, flow cytometry, and platelet-mapping techniques may provide complementary information when available, but they interrogate different aspects of platelet biology and should not be considered interchangeable [60,61].

Acquired von Willebrand abnormalities may further impair platelet adhesion through loss of high-molecular-weight vWF multimers under high shear [32,62]. Importantly, vWF antigen concentration, functional activity, and high-molecular-weight vWF multimers distribution represent distinct measures and should not be conflated.

Plasma fibrinogen concentration provides quantitative information, whereas fibrin-based VET measures assess the contribution of fibrin to whole-blood clot firmness; these measurements are therefore complementary rather than interchangeable [57,63,64].

PT/INR primarily reflects extrinsic and common-pathway coagulation activity and has limited utility for UFH monitoring. Abnormal results should be interpreted in relation to potential coagulation-factor deficiency, liver dysfunction, vitamin K status, hemodilution, and other relevant clinical circumstances rather than prompting reflexive modification of UFH [12,43].

#### 6.2.4. Monitoring Direct Thrombin Inhibitor Effect

Among direct thrombin inhibitors, bivalirudin is the principal DTI addressed in this review and is increasingly used as an alternative to UFH in selected pediatric ECMO patients [65]. Its antithrombin-independent inhibition of circulating and clot-bound thrombin may be particularly relevant when UFH is problematic because of heparin resistance, antithrombin deficiency, or heparin-induced thrombocytopenia. Renal function, flow conditions, low-flow or stagnant states, and interpatient variability may influence its anticoagulant effect [66,67].

Monitoring remains challenging. aPTT is widely used but is influenced by coagulation-factor concentrations, inflammation, inhibitors, heparin contamination, reagent characteristics, and platform-specific factors, and no universal assay or therapeutic range has been established [43,67].

Dilute thrombin time (dTT), including modified assays based on plasma dilution, provides an alternative measure of DTI effect and may offer greater analytical sensitivity and less susceptibility to some of the limitations of aPTT in selected settings [67,68]. Pediatric mechanical circulatory support data demonstrate substantial discordance between aPTT and bivalirudin-calibrated dTT. In one pediatric ECLS study, dTT correlated strongly with ecarin chromogenic testing, whereas substantial discordance was observed between dTT and heparinase-modified aPTT [68]. A subsequent pediatric VAD and ECMO cohort further evaluated bivalirudin-calibrated dTT [69]. These findings support dTT as a potentially useful complementary assay but do not establish an optimal therapeutic range or demonstrate outcome benefit from dTT-guided therapy.

Ecarin-based assays provide a mechanistically specific approach to assessing direct thrombin inhibition but have limited availability, methodological variability, and limited pediatric ECMO outcome data [43,67,68].

Accordingly, when bivalirudin is used, the monitoring strategy should be selected according to local laboratory capability and assay characteristics. Analytical performance or correlation with drug concentration should not be equated with clinical outcome benefit, and current pediatric ECMO evidence does not establish a preferred DTI assay or therapeutic range [43].

### 6.3. Integrated Hemostatic Patterns

The added value of the IHN framework is not simply the use of multiple monitoring modalities, but the interpretation of their relationships within a dynamic biological system. No individual test provides a comprehensive representation of hemostasis during ECMO; laboratory, functional, clinical, and circuit findings should therefore be interpreted as interconnected signals rather than isolated abnormalities. Table 3 illustrates this distinction by contrasting an assay-centered approach with an integrated interpretation based on concordance, discordance, underlying mechanisms, clinical context, and temporal trajectory. The table is intended as a conceptual framework for interpretation rather than a validated diagnostic or treatment algorithm.

### 6.4. Discordance Among Anticoagulation Assays

Discordance among ACT, aPTT, and anti-Xa may provide clinically useful information because these assays are influenced by different biological and analytical factors. aPTT may be affected by coagulation-factor abnormalities, consumption, hemodilution, developmental hemostasis, inflammation, inhibitors, acute-phase changes, and reagent- or platform-specific characteristics. Anti-Xa is more directly related to UFH activity but depends on antithrombin availability and assay methodology and may be affected by hemolysis, hyperbilirubinemia, hypertriglyceridemia, and preanalytical factors [12,43,47,50,51,52,53].

Discordant results should therefore not automatically be interpreted as inadequate or excessive UFH effect or used as a reflexive trigger for anticoagulant adjustment. Instead, discordance should prompt consideration of its biological and analytical determinants and correlation with other measures of hemostatic competence, clinical manifestations, and circuit findings. Anticoagulation assays remain in-vitro surrogates and do not reproduce the full interaction among blood, endothelium, inflammation, and the extracorporeal circuit.

### 6.5. Dynamic Integrated Monitoring

Integrated monitoring should be guided by the clinical trajectory rather than predefined laboratory targets. New or worsening bleeding, thrombosis, circuit dysfunction, major procedures, changes in anticoagulant requirements, hemolysis, or evolving organ dysfunction should prompt reassessment. Interpretation should account for developmental stage, recent surgery or cardiopulmonary bypass, ECMO configuration, circuit-to-patient size, and changes in the underlying disease [12,13,45,47].

Serial assessment is important because a single measurement provides only a cross-sectional description of a dynamic system. Changes over time, concordance or discordance among measurements, and new clinical or circuit findings may provide information not captured by an isolated result. Laboratory and viscoelastic testing (VET) findings should be interpreted alongside platelet and fibrinogen status and the clinical and circuit trajectory [12,13].

Integrated monitoring does not require routine measurement of every IHN component. Its principal value is interpretative: available measurements are considered as interconnected signals within the same biological system, with reassessment guided by the evolving clinical and circuit context.

## 7. Precision Hemostatic Management During Pediatric ECMO

Precision hemostatic management translates the integrated assessment of the prevailing hemostatic state into mechanism-directed intervention. Rather than correcting laboratory abnormalities in isolation, therapeutic decisions should target clinically relevant disturbances while considering their potential effects on bleeding, thrombosis, and circuit integrity.

The therapeutic approach should address the mechanisms identified through integrated assessment, with the choice and intensity of intervention determined by the prevailing hemostatic state and the anticipated benefits and risks of treatment.

The principal therapeutic domains are:

1. Anticoagulant selection and intensity;
2. Targeted blood-component and hemostatic therapy;
3. Transfusion stewardship;
4. Circuit-directed management;
5. Management of systemic drivers of dyshemostasis;
6. Procedural and Surgical Source Control;
7. Minimization of iatrogenic hemostatic burden; and
8. Serial reassessment and therapeutic adaptation.

These domains are complementary rather than sequential. Their relative importance may change as the patient’s biological condition, circuit status, anticoagulant effect, and clinical manifestations evolve.

### 7.1. Anticoagulant Selection and Intensity

#### 7.1.1. Unfractionated Heparin Anticoagulation

Unfractionated heparin (UFH) remains the conventional first-line anticoagulant during pediatric ECMO. Anticoagulation aims to limit clinically consequential patient and circuit thrombosis while minimizing bleeding. Its intensity should be individualized through integrated interpretation of anticoagulation assays, the clinical hemostatic state, and patient- and circuit-related factors rather than determined by a single laboratory value [12,43].

When clinically significant bleeding develops, anticoagulant intensity should be reassessed in relation to bleeding severity and source, circuit function, and competing thrombotic risk. Conversely, progressive thrombosis or circuit thrombogenicity should prompt reassessment of anticoagulant effect and potentially reversible contributors before anticoagulation is intensified [12,43].

The commonly used ELSO reference framework includes an anti-Xa range of approximately 0.30–0.70 U/mL and an activated partial thromboplastin time (aPTT) range of approximately 60–90 s [12]. These values should be regarded as reference ranges rather than universally validated pediatric therapeutic targets. The absence of a universal target reflects developmental and clinical heterogeneity, differences in ECMO indications and circuit conditions, and biological and analytical variability among anticoagulation assays. Available pediatric evidence has not established that a single laboratory target consistently optimizes bleeding and thrombotic outcomes across these diverse clinical settings [43,70].

Developmental stage is particularly relevant to anticoagulation because age-dependent differences in endogenous anticoagulant pathways, coagulation-factor concentrations, and the interpretation of conventional coagulation tests may influence assessment of UFH effect. These differences do not, however, establish age-specific UFH dosing adjustments or therapeutic targets. The principal developmental and clinical modifiers relevant to anticoagulation are summarized in Table 4.

The interpretation of UFH monitoring requires consideration of the biological and analytical limitations of each assay. Anti-Xa activity, aPTT, activated clotting time, and viscoelastic testing provide different, partially overlapping information and are not interchangeable. Discordance between measurements should prompt assessment of preanalytical factors, assay characteristics, antithrombin availability, coagulation-factor concentrations, inflammation, hemolysis, and other patient-specific influences before treatment is adjusted [12,43,47,50,51,52,53].

When anticoagulation measurements are discordant, their interpretation should account for the biological processes measured by each assay and the limitations of the available methods. For example, a low anti-Xa result accompanied by a prolonged aPTT does not necessarily indicate insufficient UFH effect. Antithrombin availability, coagulation-factor concentrations, inflammation, hemolysis, and assay-specific interference may contribute to this pattern. Similarly, progressive circuit thrombosis despite apparently adequate anticoagulation should prompt evaluation of flow, stasis, cannula configuration, and mechanical integrity rather than automatic escalation of UFH [12,43,47,50,51,52,53]. The clinical application of these principles requires integrating anticoagulation measurements with the patient’s hemostatic state and circuit findings. Table 5 summarizes clinical contexts that should prompt reassessment of UFH anticoagulation intensity and the key considerations to address before modifying treatment.

Anticoagulation is one modifiable component of the Integrated Hemostatic Network (IHN), not a substitute for addressing other contributors to dyshemostasis. Decisions about UFH intensity should therefore be integrated with assessment of platelet-related abnormalities, fibrinogen depletion, fibrinolytic disturbances, endothelial injury, hemolysis, inflammation, and circuit-related thrombogenicity [12,43].

#### 7.1.2. Direct Thrombin Inhibitors

UFH remains the conventional first-line anticoagulant during pediatric ECMO, but intravenous direct thrombin inhibitors (DTIs), particularly bivalirudin, provide an alternative when UFH is unsuitable or when an appropriate UFH effect cannot be reliably achieved or interpreted. Bivalirudin directly inhibits circulating and clot-bound thrombin independently of antithrombin, distinguishing it pharmacologically from UFH. This mechanism may be relevant in selected patients with impaired UFH responsiveness associated with reduced antithrombin activity. Bivalirudin may also be considered in patients with suspected or confirmed heparin-induced thrombocytopenia or clinically important heparin resistance despite evaluation of reversible causes [65].

Observational pediatric studies suggest that bivalirudin can provide effective anticoagulation during ECMO, although the available evidence remains heterogeneous [43]. A recent systematic review and meta-analysis of 12 studies involving 770 pediatric patients found that bivalirudin was associated with lower odds of major bleeding (OR 0.20, 95% CI 0.12–0.32), systemic thrombosis (OR 0.42, 95% CI 0.23–0.75), neurologic events (OR 0.47, 95% CI 0.29–0.78), and in-hospital mortality (OR 0.57, 95% CI 0.41–0.79), whereas circuit thrombosis, on-ECMO mortality, and time within the therapeutic anticoagulation range did not differ significantly from UFH [71]. However, these findings require cautious interpretation because the evidence was predominantly observational and was graded as low or very low certainty. A pediatric randomized pilot trial reported similar time within the prespecified therapeutic anticoagulation range and similar bleeding outcomes, but circuit changes for thrombosis occurred in 6 of 16 patients receiving bivalirudin versus none of 14 receiving heparin [72]. Taken together, these findings support bivalirudin as a potential alternative to UFH in selected patients but do not establish its superiority or justify its routine preferential use.

Dosing and monitoring remain incompletely standardized. Bivalirudin requirements may vary with age, renal function, ECMO configuration and indication, flow conditions, and clinical trajectory, while concomitant Continuous Renal Replacement Therapy (CRRT) may further modify drug disposition and dosing requirements [73,74,75]. Low-flow or stagnant circuit conditions may also alter the effective anticoagulant environment and should be considered when interpreting apparent changes in anticoagulant effect [65,66]. The absence of a universally accepted pediatric ECMO dosing regimen or therapeutic target reflects the limited pediatric evidence defining the relationship between bivalirudin exposure, laboratory monitoring results, and clinical outcomes; substantial variability in patient characteristics and circuit conditions; and the lack of standardized monitoring strategies validated across pediatric ECMO populations [43,73,74,75].

Switching from UFH to a DTI does not correct the broader disturbances of the IHN. Platelet dysfunction, acquired von Willebrand abnormalities, fibrinogen depletion, altered fibrinolysis, endothelial injury, hemolysis, inflammation, and circuit thrombogenicity may persist despite effective thrombin inhibition. DTI therapy should therefore be interpreted as modification of the anticoagulant component of the IHN, with anticoagulant-effect measurements considered alongside other hemostatic, clinical, and patient- and circuit-level findings.

#### 7.1.3. Antithrombin

Antithrombin (AT) is a major endogenous anticoagulant that inhibits thrombin and factor Xa. Its anticoagulant activity is markedly enhanced by heparin; therefore, unfractionated heparin (UFH) responsiveness depends partly on AT availability and functional activity [76,77].

AT activity is physiologically lower in neonates and young infants and may be further reduced during ECMO through hemodilution, consumption, reduced synthesis, or redistribution [25,78,79]. Low AT should therefore be interpreted as a potential modifier of UFH responsiveness rather than as an independent indication for replacement.

Current evidence does not support routine AT supplementation solely to normalize AT activity. The PEACE consensus found insufficient evidence to recommend routine AT monitoring or replacement in pediatric ECMO [77]. Supplementation may be considered in selected patients with persistently inadequate UFH responsiveness when reduced AT activity plausibly contributes, after evaluating anticoagulation assay validity, sampling issues, UFH administration, and other reversible causes of apparent heparin resistance. The decision should incorporate the clinical and circuit trajectory, UFH requirements, and competing bleeding and thrombotic risks [77,80].

The response to AT supplementation is variable in critically ill children. The increase in plasma AT activity depends on baseline AT activity, body weight, disease state, and extracorporeal support; consequently, a given dose may produce different increments in AT activity across patients [78,79]. This variability limits the predictability of weight-based dosing and supports reassessment of AT activity and UFH effect when supplementation is administered. A low AT value alone does not establish a need for replacement, and the available evidence does not establish that routine supplementation improves clinically important outcomes [77,80].

AT should therefore be regarded as one modifiable determinant of UFH responsiveness within the Integrated Hemostatic Network (IHN), rather than as an isolated therapeutic target. Increasing AT activity will not necessarily correct thrombosis when platelet activation, inflammation, endothelial injury, impaired fibrinolysis, blood–surface interactions, or circuit-related stasis also contribute to the process.

### 7.2. Targeted Blood-Component and Hemostatic Therapy

Blood-component and factor replacement therapy should be directed toward achieving adequate hemostatic competence rather than normalizing isolated laboratory values. Therapeutic decisions should be guided by the prevailing clinical hemostatic state, interpreted in the context of the clinical indication, procedural requirements, and the anticipated effects of treatment on the overall hemostatic balance. The principal therapeutic options and clinical considerations are summarized in Table 6.

#### 7.2.1. Platelet Therapy

Platelet transfusion should be considered when thrombocytopenia or clinically relevant platelet dysfunction contributes to impaired hemostatic competence. The PEACE consensus suggests considering maintenance of platelet counts above 100 × 10^9^/L in three specific contexts: postcardiotomy ECMO during the periprocedural period, high-bleeding-risk invasive procedures, and clinically significant bleeding [45,47]. These recommendations are based on low- or very-low-quality pediatric evidence and should be individualized according to the severity and trajectory of bleeding, developmental stage, recent surgery or cardiopulmonary bypass, procedural risk, and other evidence of impaired hemostasis.

In nonbleeding patients outside these defined contexts, platelet transfusion thresholds vary among pediatric ECMO programs, and no universally validated threshold has been established [81,82]. This uncertainty reflects the limited evidence linking specific platelet-count thresholds to improved clinical outcomes. A universally validated platelet transfusion threshold has not been established for pediatric ECMO because available studies provide limited evidence linking specific platelet-count thresholds to clinically meaningful outcomes. Moreover, platelet count alone does not capture platelet function or overall clot strength, and transfusion requirements vary with bleeding status, developmental stage, procedural context, and competing thrombotic risk. The ECSTATIC study evaluated the feasibility of comparing two prophylactic platelet transfusion thresholds but was not designed to establish the efficacy or safety of either strategy [83]. Accordingly, platelet count should inform, but not independently determine, transfusion decisions in nonbleeding pediatric ECMO patients.

#### 7.2.2. Plasma Therapy

In patients with clinically significant bleeding or an anticipated high-risk procedure, plasma may be considered when a clinically relevant deficiency of multiple coagulation factors is suspected or demonstrated and contributes to bleeding or increased bleeding risk. In patients with clinically relevant bleeding, the PEACE consensus considers an initial plasma transfusion reasonable when the INR is greater than 1.5 [45]. This recommendation should be interpreted in the clinical context, as an isolated prolonged INR does not necessarily establish a clinically relevant coagulation-factor deficiency. INR may also be influenced by anticoagulant effect, developmental hemostasis, liver dysfunction, vitamin K deficiency, factor consumption or dilution, and assay-related factors. Repeated plasma transfusions solely to correct the INR may not improve outcomes [45]. Plasma should therefore be used to address clinically relevant coagulation-factor deficiency rather than to normalize an isolated laboratory abnormality.

#### 7.2.3. Fibrinogen Replacement

Fibrinogen replacement addresses a distinct component of hemostatic competence. Fibrinogen concentrate or cryoprecipitate may be considered when reduced fibrinogen concentration or impaired functional fibrin contribution contributes to clinically significant bleeding or is relevant to a high-risk procedure [77]. Fibrinogen concentrate provides a more standardized fibrinogen dose, whereas cryoprecipitate provides a variable amount of fibrinogen together with additional hemostatic proteins, including factor VIII and von Willebrand factor [84].

For pediatric ECMO patients after cardiopulmonary bypass and those undergoing high-bleeding-risk procedures, the PEACE consensus suggests considering fibrinogen levels above 150 mg/dL during the periprocedural period. In the setting of periprocedural bleeding, higher transfusion thresholds may be maintained until bleeding ceases or decreases to minimal or moderate severity [47]. These are weak recommendations based on low- or very-low-quality pediatric evidence and should not be interpreted as a universal therapeutic threshold.

A universally validated fibrinogen threshold has not been established for pediatric ECMO because pediatric evidence linking specific fibrinogen concentrations to bleeding control, thrombotic complications, or other clinically meaningful outcomes remains limited. In addition, fibrinogen concentration does not fully capture functional fibrin contribution to clot formation, and the relevance of a given value depends on bleeding severity, procedural context, developmental stage, and the broader hemostatic state. The available periprocedural recommendations should therefore be interpreted as context-specific guidance rather than as a universal transfusion threshold.

The optimal fibrinogen target remains uncertain and should be individualized according to bleeding severity and trajectory, procedural risk, and available functional assessment [12,77].

#### 7.2.4. Red Blood Cell Transfusion

RBC transfusion should be guided by the clinical context and adequacy of oxygen delivery rather than hemoglobin concentration alone. Relevant considerations include hemodynamic status, ongoing blood loss, oxygen delivery and consumption, ECMO support, and the patient’s clinical trajectory.

In hemodynamically stable critically ill children without active bleeding or evidence of inadequate oxygen delivery, restrictive transfusion strategies using a hemoglobin threshold of approximately 7 g/dL have been supported by randomized pediatric critical care data and subsequent consensus recommendations [85,86]. However, these major trials excluded pediatric ECMO patients. Pediatric ECMO-specific observational data also suggest that transfusion practice can be restrictive in selected patients, but do not establish a universal hemoglobin threshold or demonstrate an outcome benefit for a specific transfusion strategy [87,88]. Accordingly, during pediatric ECMO, hemoglobin concentration should not be the sole trigger for RBC transfusion. Decisions should reflect the clinical scenario and adequacy of systemic or regional oxygen delivery, considered alongside hemoglobin concentration; ongoing bleeding, ECMO mode and indication, and the patient’s evolving clinical course provide important context. Current evidence does not establish a single hemoglobin threshold for RBC transfusion in pediatric ECMO [81].

#### 7.2.5. Rescue Hemostatic Therapies

PCC and rFVIIa should be reserved for exceptional rescue in refractory, life-threatening bleeding after source control and conventional multimodal therapy. Pediatric ECMO evidence remains limited, and the potential for patient and circuit thrombosis should be considered [45,89]. Neither should be used simply to correct an abnormal coagulation test in the absence of clinically significant bleeding.

#### 7.2.6. Antifibrinolytic Therapy

Tranexamic acid or ε-aminocaproic acid may be considered in patients with clinically significant bleeding or those anticipated to be at high risk of bleeding, particularly in surgical or high-risk procedural settings, as part of a multimodality blood-management strategy [90]. Evidence or clinical suspicion of excessive fibrinolysis may provide additional support for treatment, and VET findings may contribute when available. The potential for thrombotic complications should be considered, particularly in patients with active or progressive patient or circuit thrombosis.

### 7.3. Transfusion Stewardship and Transfusion as a Biological Perturbation

Transfusion may be lifesaving during active hemorrhage, clinically significant anemia, or clinically relevant hemostatic deficits. However, transfusion requirements should be interpreted in relation to the underlying hemostatic disturbance. In pediatric ECMO, transfusion exposure is common and substantial, while optimal thresholds remain incompletely defined.

Observational studies in pediatric and adult ECMO populations have reported associations between greater RBC, plasma, or platelet transfusion exposure and adverse outcomes, including mortality, bleeding, and thrombosis [91,92,93,94]. These associations do not establish causality because transfusion exposure is strongly influenced by illness severity, bleeding, surgery, ECMO duration, and other clinical factors.

Transfused components may modify the intravascular hemostatic and inflammatory environment. During storage, RBCs undergo metabolic and membrane changes, while platelets may develop storage-related changes in activation, function, and membrane integrity. These changes may alter the biological effects of transfused components; however, their specific clinical consequences during pediatric ECMO remain incompletely defined [95,96].

Platelet transfusion exposure has been associated with subsequent bleeding and thrombosis in pediatric ECMO cohorts, and platelet transfusion has also been associated with changes in oxygenator function in selected patients [92,97]. These associations highlight the complex relationship between transfusion exposure, the underlying hemostatic disturbance, and circuit behavior during ECMO.

Transfusion stewardship should therefore focus on indication, timing, dose, and reassessment, rather than on minimizing transfusion irrespective of clinical need. Each transfusion should address a defined physiological or hemostatic problem, followed by reassessment of its intended effect and consideration of whether additional exposure is justified.

### 7.4. The ECMO Circuit as an Active Therapeutic Target

The ECMO circuit should be regarded as an active component of the hemostatic environment rather than merely as a life-support device. Continuous blood exposure to artificial surfaces and nonphysiological flow conditions can promote platelet and contact activation, complement activation, inflammation, hemolysis, and shear-mediated blood trauma. Abnormal flow, stasis, excessive shear, and cannula-related disturbances may further promote thrombin generation and fibrin deposition. These effects may persist despite systemic anticoagulation, while progressive circuit thrombosis may amplify consumption and mechanical blood trauma. The circuit can therefore function both as a source and an amplifier of dyshemostasis within the IHN [98,99].

Circuit management should address cannula configuration and position, blood flow and drainage, pump conditions, and mechanical integrity while minimizing unnecessary blood–biomaterial exposure. Circuit-related findings should be interpreted alongside anticoagulant effect, platelet and fibrinogen status, hemolysis, patient condition, and the trajectory of circuit performance. These elements provide a framework for identifying potentially modifiable circuit-related contributors to hemostatic disturbances and prioritizing targeted corrective measures. Table 7 summarizes circuit-based strategies for hemostatic optimization during pediatric ECMO.

Circuit findings should therefore be interpreted as part of the evolving hemostatic process rather than as isolated indicators of anticoagulant adequacy. Progressive fibrin deposition, increasing oxygenator pressure gradients, flow instability, recurrent suction events, or increasing hemolysis may indicate evolving circuit pathology. Circuit or oxygenator exchange should be considered when abnormalities become clinically consequential, particularly when associated with worsening oxygenator function, significant hemolysis, inability to maintain adequate flow, or compromised patient support. Because exchange creates a new blood–biomaterial interface, its expected clinical benefit should outweigh the additional biological and procedural burden [98].

Importantly, circuit thrombosis should not automatically be interpreted as inadequate systemic anticoagulation. Before increasing UFH intensity, clinicians should assess whether anticoagulant effect is adequate and whether mechanical contributors are present, including low flow or stasis, cannula malposition or drainage obstruction, excessive shear, recurrent suction, or other circuit abnormalities.

A thrombotic circuit finding may therefore reflect inadequate anticoagulation, a mechanical problem, blood–surface interactions, excessive shear, inflammation, or several interacting mechanisms. Increasing systemic anticoagulation without addressing a reversible mechanical contributor may fail to correct the underlying disturbance while potentially increasing bleeding risk. Circuit management is consequently an upstream and potentially reversible component of precision hemostasis, complementary to rather than a substitute for anticoagulation and targeted hemostatic therapy.

### 7.5. Management of Systemic Drivers of Dyshemostasis

Hemostatic management cannot be separated from the physiological conditions in which hemostasis operates. Hypothermia, acidosis, hypocalcemia, shock, severe inflammation, infection, hepatic or renal dysfunction, ongoing tissue injury, and impaired perfusion may alter hemostatic pathways and modify the effects of anticoagulant and hemostatic therapies [45,100].

These abnormalities should be regarded as contributors to dyshemostasis rather than unrelated supportive-care issues. However, the management of systemic physiological disturbances should remain proportionate to the clinical problem: the objective is not to normalize every physiological variable solely because it may influence hemostasis, but to maintain conditions compatible with survival, adequate organ function, and recovery while addressing reversible disturbances that may contribute to bleeding, thrombosis, impaired circuit function, or altered responses to therapy.

### 7.6. Procedural and Surgical Source Control

Local and surgical control of bleeding is an integral component of hemostatic management during ECMO. Cannulation-site bleeding, postoperative bleeding, vascular-access bleeding, chest-tube losses, and procedure-related hemorrhage may persist despite correction of systemic abnormalities when a structural source remains uncontrolled [47,100]. Unnecessary invasive procedures should therefore be avoided whenever clinically feasible, and the indication, timing, and bleeding risk of necessary procedures should be considered before intervention [47].

When clinically significant bleeding is temporally related to a procedure or surgical source, temporary reduction or suspension of systemic anticoagulation may be considered while definitive source control is pursued, provided the competing risk of patient or circuit thrombosis is assessed [47]. Perioperative anticoagulation management should therefore be individualized according to the severity and source of bleeding, procedural urgency and bleeding risk, current hemostatic state, ECMO configuration and circuit condition, and thrombotic risk.

Whenever feasible, definitive source control should be pursued concurrently with correction of clinically relevant systemic hemostatic deficits. Decisions regarding anticoagulation interruption, transfusion, fibrinogen replacement, and antifibrinolytic therapy should be individualized according to the procedural context and the evolving bleeding and thrombotic condition of the patient and circuit.

### 7.7. Minimization of Iatrogenic Hemostatic Burden

Minimizing iatrogenic hemostatic burden is an integral component of precision management. Anticoagulation, transfusion, invasive procedures, repeated blood sampling, and circuit manipulation may each be clinically necessary but may also introduce additional bleeding, thrombotic, or procedural risk. This is particularly relevant in pediatric ECMO because of limited circulating blood volume, substantial circuit-to-patient volume ratios, and repeated exposure to invasive interventions [100].

The potential contribution of each intervention to cumulative iatrogenic burden should therefore be considered alongside its expected clinical benefit. Practical strategies include limiting unnecessary blood sampling, avoiding unnecessary procedures, planning necessary interventions according to bleeding and thrombotic risk, reviewing medications that may impair platelet or coagulation function, avoiding unnecessary transfusion, and minimizing avoidable circuit manipulation.

The objective is not to withhold necessary interventions, but to ensure that each intervention has a defined clinical purpose, that potential harms are considered, and that its effects are reassessed within the evolving IHN.

### 7.8. Serial Reassessment and Therapeutic Adaptation

Precision hemostatic management is a dynamic, iterative process rather than a sequence of fixed interventions. The clinical hemostatic state, laboratory profile, circuit condition, and underlying physiological drivers may change rapidly, and an intervention directed at one component of the IHN may modify others [43,44].

Therapeutic decisions should therefore be revisited after clinically meaningful changes in bleeding, thrombogenicity, anticoagulant effect, transfusion exposure, circuit performance, systemic physiology, or procedural status. Reassessment should determine whether the intended effect was achieved and whether the mechanisms contributing to dyshemostasis remain active, have evolved, or have become reversible [43,44,45,47].

Persistent bleeding despite correction of an apparent coagulation abnormality should prompt reconsideration of the underlying mechanism rather than automatic escalation of hemostatic therapy. Surgical or procedural bleeding, platelet dysfunction, fibrinolytic abnormalities, anticoagulation intensity and its concordance with the intended therapeutic target, circuit-related injury, and systemic drivers should be reconsidered as potential contributors. Conversely, new or progressive thrombosis should prompt reassessment of anticoagulant effect and potentially reversible patient- or circuit-related contributors before intensifying anticoagulation [44,45].

Therapeutic adaptation should follow the evolving clinical and biological context, with treatment escalated, de-escalated, redirected, or discontinued according to the observed response and the mechanisms that remain clinically relevant.

### 7.9. Toward Precision Hemostasis

The objective of precision hemostasis is not to normalize laboratory values or eliminate every biological response to extracorporeal support, but to achieve sufficient coordinated hemostatic function to control clinically relevant bleeding, limit patient and circuit thrombosis, preserve circuit integrity, and minimize treatment-related iatrogenic burden. This requires consideration of clinical manifestations, anticoagulant effect, platelet and fibrinogen contribution, functional hemostatic assessment, circuit condition, developmental and disease context, and potentially reversible systemic or mechanical contributors [12,13,29,44,98,100].

The IHN framework provides a conceptual and testable platform for integrating developmental, biological, laboratory, functional, clinical, and circuit determinants and translating their relationships into mechanism-directed interventions and sequential reassessment. Its potential clinical application includes structured assessment of dyshemostasis, identification of reversible mechanisms, selection of targeted interventions, and adaptation of therapy according to the evolving clinical and biological context. Although the existing literature supports the biological rationale for integrated assessment and multimodal monitoring, the proposed systems-level integration has not been validated as a clinical decision-making algorithm. Prospective studies are needed to determine whether this approach improves clinical decision-making and patient outcomes.

## 8. Current Evidence and Limitations

Evidence supporting hemostatic management in pediatric extracorporeal membrane oxygenation (ECMO) remains limited and heterogeneous. The literature is dominated by retrospective observational studies, single-center cohorts, and studies with variable definitions of bleeding, thrombosis, and anticoagulation-related outcomes. In a systematic literature review, Drop et al. identified substantial heterogeneity in study populations, ECMO circuits, anticoagulation protocols, monitoring practices, and transfusion triggers, with conflicting findings across several domains of hemostatic management [101]. More recently, a systematic review and meta-analysis of pediatric ECMO studies identified 69 distinct bleeding definitions, demonstrating how differences in outcome ascertainment can affect reported bleeding prevalence and limit comparisons across studies [102]. Consequently, findings from individual cohorts and pooled analyses should be interpreted in light of their methodological limitations and the clinical heterogeneity of pediatric ECMO populations.

To provide an overview of the evidence informing current practice, Table 8 summarizes principal studies, systematic reviews, and consensus recommendations across five clinically relevant domains: bleeding and overall hemostatic complications; hemostatic monitoring; hemostatic factor replacement, blood component transfusion, and adjunctive therapies; anticoagulation with bivalirudin versus unfractionated heparin; and antithrombin supplementation. Evidence sources are grouped by clinical domain rather than ranked solely by study design, because consensus recommendations and observational studies often inform decisions in areas where comparative trials are unavailable. The table also distinguishes direct pediatric evidence from findings extrapolated from adult ECMO populations and highlights the limitations that constrain clinical interpretation.

The pediatric context is particularly important when interpreting this evidence. Developmental hemostasis, body size, circulating blood volume, the circuit-to-patient blood-volume relationship, preceding cardiopulmonary bypass, concurrent continuous renal replacement therapy, and disease-specific factors may modify both hemostatic biology and the interpretation of laboratory measurements. These factors complicate the extrapolation of adult ECMO findings to neonates and children. The systematic review by Drop et al. [101] identified substantial variation in the populations and clinical practices represented in pediatric studies, reinforcing the need for prospective multicenter research with standardized outcome definitions and more consistent reporting. Blood-sampling burden and the feasibility of repeated testing are additional considerations when evaluating monitoring strategies in children.

Evidence supporting anticoagulation and functional hemostatic monitoring is similarly heterogeneous. Activated clotting time (ACT), activated partial thromboplastin time (aPTT), anti-factor Xa assays, and viscoelastic testing (VET) interrogate different aspects of anticoagulant effect or hemostatic function and are subject to distinct biological, analytical, and preanalytical limitations. Drop et al. documented a lack of standardized reporting of coagulation testing and related variables in pediatric ECMO studies [103]. The Pediatric Extracorporeal Membrane Oxygenation Anticoagulation CollaborativE (PEACE) consensus recommendations address the interpretation and use of anticoagulation monitoring assays and therapeutic targets in this population [43]. However, the available evidence does not establish a single optimal monitoring strategy or universally applicable therapeutic ranges. Pediatric VET studies also vary in platforms, reagents, sampling conditions, reference ranges, and clinical endpoints [55,56,57,58,59]. VET should therefore be considered complementary to conventional laboratory tests and clinical assessment rather than a validated standalone guide to anticoagulation or hemostatic therapy.

Similar uncertainty applies to transfusion and factor-replacement thresholds. PEACE recommendations provide a structured framework for prophylactic blood component transfusion in neonates and children receiving ECMO, but they are based on a systematic-review-informed consensus process rather than definitive comparative trials establishing the superiority of specific thresholds [80]. The same distinction is important when considering replacement of antithrombin, fibrinogen, and von Willebrand factor, as well as antifibrinolytic agents and other adjunctive hemostatic therapies [89]. Although consensus recommendations can guide clinical decisions, the evidence remains insufficient to define universally applicable replacement thresholds or support routine use of every intervention in all patients.

This limitation is particularly relevant to platelet transfusion. The ECSTATIC feasibility trial prospectively evaluated the feasibility of comparing restrictive and liberal prophylactic platelet transfusion thresholds in children receiving ECMO [82]. However, the study was designed primarily to assess feasibility and was not powered to establish the efficacy, safety, or superiority of a specific platelet threshold. Accordingly, its findings should not be interpreted as definitive evidence in favor of either strategy. Decisions about platelet and fibrinogen replacement must still account for active bleeding, procedural requirements, developmental stage, and the overall clinical hemostatic state.

Evidence concerning antithrombin supplementation and other factor-directed interventions is also inconclusive. In a systematic review and meta-analysis of ECMO studies, Elliott et al. found no statistically significant overall differences in bleeding, thrombotic events, or mortality associated with antithrombin supplementation; however, the pediatric subgroup showed an association between supplementation and increased thrombotic events [80]. These findings cannot establish causality because of potential confounding by indication and residual confounding, but they argue against assuming that laboratory correction or routine supplementation necessarily improves clinical outcomes. Evidence for antifibrinolytic therapy, prothrombin complex concentrate, recombinant activated factor VII, and other factor-directed interventions remains limited; their use should be guided by the suspected mechanism of dyshemostasis, developmental stage, procedural context, and competing thrombotic risk [45,47,89].

Uncertainty also persists regarding the comparative effectiveness of anticoagulant agents. Although unfractionated heparin remains widely used, direct thrombin inhibitors such as bivalirudin have increasingly been investigated as alternatives. A pediatric systematic review and meta-analysis published in 2026 reported associations between bivalirudin use and lower odds of major bleeding and systemic thrombosis compared with heparin, but the evidence base consisted predominantly of observational studies [71]. These pooled associations are clinically relevant but do not establish causal benefit or demonstrate the superiority of bivalirudin, particularly given potential confounding by indication, differences in anticoagulation protocols, and heterogeneity in outcome definitions. Larger prospective multicenter studies and adequately powered randomized trials are needed to establish comparative effectiveness and safety.

Bleeding and thrombotic complications remain incompletely characterized in pediatric ECMO [10,102]. Variability in bleeding definitions may contribute to differences in reported bleeding prevalence. Similarly, the detection of patient thrombosis depends on the clinical indications for imaging, surveillance practices, availability of diagnostic modalities, and the ability to distinguish patient thrombosis from circuit fibrin deposition, circuit thrombosis, and oxygenator dysfunction. These sources of heterogeneity may influence both event detection and reporting, contributing to variability in reported complication rates [10]. Consequently, the absence of a clinically detected thrombotic event should not necessarily be interpreted as evidence that thrombotic activity was absent.

Finally, the bleeding-predominant, thrombosis-predominant, and mixed hemostatic states proposed within the Integrated Hemostatic Network (IHN) framework are working clinical constructs intended to organize clinical reasoning and generate testable hypotheses. Their reproducibility across clinicians and institutions, predictive value, and potential role in guiding treatment remain unknown. Although individual components of the IHN—including platelet and endothelial dysfunction, coagulation and fibrinolytic abnormalities, complement activation, inflammation, hemolysis, and circuit-related disturbances—have each been implicated in ECMO-associated dyshemostasis, their temporal relationships and relative contributions remain incompletely characterized in pediatric ECMO. The IHN should therefore be regarded as a hypothesis-generating framework whose ability to improve clinical decision-making or patient outcomes requires prospective evaluation.

## 9. Future Perspectives

The next phase of pediatric ECMO hemostasis research should move beyond isolated laboratory abnormalities toward longitudinal, multimodal characterization of dyshemostasis. The central question is whether integrated clinical, laboratory, functional, biological, and circuit information can identify clinically meaningful changes early enough to support mechanism-directed intervention and improve outcomes.

### 9.1. Define Longitudinal Trajectories of Dyshemostasis

Prospective longitudinal studies should characterize the temporal evolution of platelet, coagulation, fibrinolytic, endothelial, inflammatory, hemolytic, and circuit-related variables throughout ECMO support. Studies should incorporate developmental stage and use harmonized definitions of bleeding, thrombosis, circuit thrombosis, and oxygenator dysfunction. Such datasets could determine whether reproducible trajectories correspond to the proposed working hemostatic states and identify transitions between them.

### 9.2. Determine the Clinical Value of Multimodal Monitoring

Future studies should establish whether integrated monitoring provides earlier or more accurate recognition of clinically relevant dyshemostasis than conventional assay-based strategies. This requires evaluating whether multimodal information changes clinical decisions and, importantly, whether such changes translate into improved patient or circuit outcomes. The incremental value of VET, platelet-function assessment, DTI-specific assays, and selected biological markers should be assessed within this broader clinical context rather than as isolated tests.

### 9.3. Test Adaptive, Mechanism-Directed Treatment Strategies

The IHN framework generates a testable hypothesis that anticoagulation and hemostatic therapy could be adapted according to the evolving clinical, laboratory, functional, and circuit context rather than fixed laboratory targets alone. Prospective studies could compare conventional target-based strategies with adaptive approaches incorporating bleeding or thrombotic trajectory, assay concordance or discordance, functional hemostatic findings, and circuit status. Similar evaluation is needed for platelet and fibrinogen replacement, antithrombin supplementation, antifibrinolytic therapy, and other factor-directed interventions.

### 9.4. Validate the Systems-Level IHN Architecture

Multicenter mechanistic studies are needed to determine whether the proposed relationships among developmental, biological, laboratory, clinical, and circuit determinants are reproducible across pediatric ECMO populations. Longitudinal designs should evaluate interactions among network components and identify trajectories that are clinically meaningful and potentially actionable. Harmonized definitions, laboratory methodologies, and data collection will be essential, while analyses should account for developmental stage, ECMO configuration, underlying disease, preceding cardiopulmonary bypass, and institutional practice.

### 9.5. Define Pediatric-Specific Modifiers

Future research should explicitly examine how age, developmental hemostasis, body size, circulating blood volume, circuit-to-patient blood-volume ratio, ECMO configuration, preceding cardiopulmonary bypass, renal function, CRRT, and underlying disease modify dyshemostasis and treatment response. Pediatric feasibility is also important: blood-sampling requirements, assay turnaround time, sample volume, and the availability of specialized functional assays may substantially influence the clinical applicability of multimodal monitoring.

### 9.6. Evaluate Data Integration and Artificial Intelligence

High-dimensional longitudinal datasets may provide an opportunity to identify complex interactions and time-dependent patterns that are difficult to recognize using conventional approaches. Machine-learning and other analytical methods could be explored for this purpose, but their value should be assessed beyond predictive performance alone. Future studies should address interpretability, calibration, robustness across developmental stages and ECMO configurations, external validation, and—most importantly—whether such approaches improve clinical decisions and patient outcomes.

Ultimately, the field should progress from reactive correction of isolated laboratory abnormalities toward anticipatory, mechanism-directed precision hemostasis. Such an approach would integrate developmental and disease context, evolving clinical trajectory, anticoagulant effect, functional hemostasis, circuit condition, and selected biological signals to identify clinically important changes and support targeted therapeutic adaptation.

## 10. Conclusions

ECMO-associated dyshemostasis in children is a dynamic disturbance of an interconnected Integrated Hemostatic Network rather than a simple consequence of excessive or insufficient anticoagulation. Developmental hemostasis, blood–circuit interactions, platelet and endothelial dysfunction, coagulation and fibrinolytic pathways, complement, inflammation, hemolysis, and other biological processes interact continuously to shape the evolving hemostatic state.

Bleeding, thrombosis, and mixed manifestations represent different clinical expressions of these interacting disturbances. The proposed bleeding-predominant, thrombosis-predominant, and mixed hemostatic states provide a framework for describing this evolving clinical expression and for organizing the relationships among underlying mechanisms, clinical findings, and therapeutic considerations.

Precision hemostatic management extends beyond normalization of individual laboratory values toward mechanism-directed and dynamically reassessed intervention. Anticoagulant effect, platelet and fibrinogen contribution, functional hemostatic assessment, clinical manifestations, circuit condition, developmental and disease context, and potentially reversible systemic or mechanical contributors may each provide complementary information about the evolving hemostatic state.

The central contribution of the IHN framework is the explicit integration of these interacting determinants into a unified, dynamic, and testable systems-level architecture for pediatric ECMO. Its conceptual advance lies not simply in recognizing the multifactorial nature of dyshemostasis or in combining multiple monitoring modalities, but in framing their relationships and temporal evolution as components of a single system that can generate clinically testable hypotheses.

Future research should determine whether this integrated perspective can identify reproducible patterns of evolving dyshemostasis, support earlier recognition of clinically important changes, and enable more targeted and adaptive therapeutic strategies. Such studies may ultimately clarify whether systems-level integration can translate into better patient-centered outcomes in pediatric ECMO.

## Figures and Tables

**Table 1:** Developmental modifiers of the Integrated Hemostatic Network and their relevance to pediatric ECMO.

Domain	Developmental or Clinical Consideration	Potential Relevance to Pediatric ECMO
Coagulation factors	Concentrations of several coagulation factors vary with developmental stage. Vitamin K–dependent factors and contact-pathway factors are relatively low at birth, whereas factor VIII and von Willebrand factor are generally preserved or elevated	Alters the baseline sensitivity and reference intervals of conventional assays (PT, aPTT, and TT), potentially mimicking or masking circuit-induced factor consumption.
Endogenous anticoagulant pathways	Antithrombin and proteins C and S are physiologically reduced in neonates compared with adults, but their developmental trajectories differ: antithrombin approaches adult levels during early infancy, whereas proteins C and S mature more gradually throughout childhood. These differences contribute to the developmentally balanced neonatal hemostatic system and may influence the response to anticoagulant therapy during ECMO	Developmental differences may influence the response to anticoagulants, particularly heparin, whose activity depends on antithrombin. Anticoagulation measurements should be interpreted in the context of developmental hemostasis and assay characteristics.
Platelet biology	Platelet number is generally adequate at birth, although platelet responsiveness differs from that of adults. Higher von Willebrand factor concentrations and differences in its multimer distribution may partly compensate for reduced responses to some platelet agonists	Standard platelet counts underrepresent functional hemostatic capacity, especially as mechanical shear stress from the circuit selectively depletes high-molecular-weight vWF multimers.
Fibrinolysis	Plasminogen concentrations are relatively low at birth, while t-PA and PAI-1 levels and activity vary with developmental age. These differences may alter the balance between plasmin generation and inhibition, although their net effect on fibrinolytic capacity cannot be inferred from individual components alone	Developmental differences in fibrinolytic regulation may influence fibrin persistence and clot stability, potentially contributing to oxygenator fibrin accumulation when fibrinolysis is insufficient or to bleeding when fibrin degradation is excessive. The clinical expression depends on the interaction between developmental hemostasis, ECMO-associated coagulation and inflammatory disturbances, and anticoagulant therapy.
Patient–circuit relationship	Body size, circuit surface area, priming volume, and the ratio of extracorporeal to circulating blood volume vary with patient size	Causes profound immediate hemodilution upon cannulation—abruptly diluting clotting factors and platelets—while amplifying contact activation per unit of blood exposed.

**Table 2:** Laboratory and functional markers for precision monitoring of dyshemostasis during pediatric ECMO.

Monitoring Modality	Primary Information Provided	Important Limitations	Potential Contribution to Monitoring
Activated clotting time (ACT)	Whole-blood assessment of clot initiation; commonly used for bedside UFH monitoring	Not specific for UFH; affected by thrombocytopenia, platelet dysfunction, hypofibrinogenemia, coagulation-factor deficiencies, hypothermia, hemodilution, anemia, inflammation, and assay characteristics	Provides rapid bedside information on clot initiation but should be interpreted with complementary laboratory and clinical findings
Activated partial thromboplastin time (aPTT)	Functional assessment of intrinsic/common coagulation pathways; commonly used for UFH and bivalirudin monitoring	Strongly influenced by reagent, platform, and calibration; affected by coagulation-factor deficiencies or consumption, inflammation, liver dysfunction, hemodilution, inhibitors, and developmental hemostasis	Provides an estimate of anticoagulant effect but requires assay-specific interpretation and correlation with other measures
Anti-factor Xa	Relatively direct estimate of UFH activity through inhibition of factor Xa	Depends on antithrombin availability and assay methodology; may be affected by hemolysis, hyperbilirubinemia, hypertriglyceridemia, and preanalytical or platform-specific factors; does not assess global hemostasis	Helps characterize UFH effect but should not be interpreted as a measure of overall hemostatic competence
Heparin concentration	Estimate of circulating heparin exposure	Does not necessarily reflect biological anticoagulant effect; methods may not be interchangeable	May provide complementary information when anticoagulation assays are discordant
Viscoelastic testing (VET; TEG/ROTEM)	Dynamic whole-blood assessment of clot initiation, propagation, strength, stability, and lysis	Pediatric ECMO thresholds are not standardized; results depend on platform, reagents, sampling and preanalytical conditions, developmental hemostasis, and reproducibility	Provides functional information on clot formation, platelet–fibrin interaction, and fibrinolysis that complements plasma-based assays
HEPTEM/heparinase-modified VET	Assesses clot initiation after neutralization of heparin effect	Interpretation requires comparison with the corresponding native assay; does not independently characterize overall hemostatic competence; pediatric ECMO-specific therapeutic thresholds are not established	May help determine whether delayed clot initiation is substantially attributable to heparin effect
FIBTEM/functional fibrinogen	Estimates the contribution of fibrin to whole-blood clot firmness	Does not independently characterize platelet contribution or overall hemostatic competence; platform-specific	Helps distinguish impaired fibrin contribution from platelet-related or other causes of reduced clot strength
VET lysis parameters	Assess clot stability and fibrinolytic activity	Influenced by platform, reagent, sampling, and analytical conditions; pediatric ECMO thresholds remain insufficiently established	May identify altered fibrinolysis not captured by conventional coagulation assays
Platelet count	Quantifies circulating platelets	Does not assess platelet function; thrombocytopenia may coexist with qualitative dysfunction	Provides quantitative information that should be interpreted in relation to potential mechanisms of platelet loss and clinical context
Platelet function testing	Assesses selected aspects of platelet activation and function	Aggregometry, flow cytometry, and platelet-mapping techniques interrogate different aspects of platelet biology and are not interchangeable; availability is limited	May identify qualitative platelet dysfunction not apparent from platelet count
Fibrinogen concentration	Quantifies circulating fibrinogen	Does not directly assess the functional contribution of fibrinogen to whole-blood clot strength	Quantifies fibrinogen concentration, complementing fibrin-based VET parameters and clinical assessment
PT/INR	Assesses extrinsic and common-pathway coagulation activity	Limited utility for UFH monitoring; affected by factor deficiency, liver dysfunction, vitamin K status, hemodilution, and assay characteristics	May identify clinically relevant coagulation-factor abnormalities rather than guide UFH dosing
dTT/plasma-diluted thrombin time	Provides complementary assessment of direct thrombin inhibitor effect, particularly when aPTT is discordant	Limited availability; calibration and methodology vary; therapeutic ranges and outcome-based targets remain insufficiently established; results may be discordant with aPTT	May provide complementary information when monitoring bivalirudin, particularly when aPTT is discordant
Bivalirudin-calibrated dTT	Drug-calibrated assessment of bivalirudin anticoagulant effect	Calibration, availability, and methodology vary; optimal pediatric ECMO therapeutic ranges and outcome-based targets remain unestablished	May provide a drug-calibrated assessment of bivalirudin effect in selected settings
Ecarin-based assays	Mechanistically specific assessment of direct thrombin inhibition	Limited availability, lack of standardization, and sparse pediatric ECMO outcome data	May provide complementary assessment of DTI effect when available
Clinical and circuit assessment	Detects bleeding, thrombosis, circuit fibrin deposition, oxygenator dysfunction, flow abnormalities, hemolysis, and evolving clinical manifestations	Context-dependent and cannot independently define the underlying hemostatic mechanism	Establishes the clinical relevance and trajectory of laboratory and functional abnormalities and distinguishes patient- from circuit-related manifestations

No individual modality provides a complete assessment of hemostasis during pediatric ECMO. Interpretation should consider developmental stage, underlying disease, ECMO configuration, recent surgery or cardiopulmonary bypass, anticoagulant exposure, clinical manifestations, and patient- and circuit-related findings. VET and DTI-monitoring approaches remain incompletely standardized in pediatric ECMO, and no single assay or numerical value has been established as a universal therapeutic target.

**Table 3:** From Multimodal Monitoring to an Integrated Hemostatic Network Approach.

Conventional Multimodal Monitoring	Integrated Hemostatic Network Approach
Combines complementary laboratory and functional assays	Interprets complementary findings as interconnected manifestations of a dynamic biological network
Focuses primarily on individual measurements and predefined targets	Focuses on relationships, concordance, discordance, and temporal trajectories across measurements
Anti-Xa or aPTT assesses anticoagulant effect	Discordance among assays prompts consideration of anticoagulant exposure, antithrombin availability, coagulation-factor abnormalities, inflammation, assay characteristics, and clinical context
Platelet count identifies thrombocytopenia	Interprets thrombocytopenia according to potential mechanisms—including consumption, hemodilution, bleeding, inflammation, sequestration, and circuit-related activation—and in relation to platelet function, vWF–endothelial interactions, anticoagulant effect, circuit findings, and clinical trajectory
Fibrinogen concentration identifies hypofibrinogenemia	Interprets fibrinogen abnormalities in relation to fibrin formation, clot strength, and fibrinolysis, while considering consumption, dilution, bleeding, inflammation, and coagulation activation
VET characterizes clot initiation, strength, and lysis	Interprets VET patterns as functional manifestations of interacting processes, such as impaired fibrin contribution, platelet dysfunction, anticoagulant effect, or combined abnormalities, in relation to clinical and circuit findings
Circuit inspection identifies fibrin deposition, thrombus formation, or other circuit abnormalities	Distinguishes patient thrombosis, circuit fibrin deposition, oxygenator dysfunction, and circuit thrombosis, and interprets each in relation to anticoagulant effect, platelet and coagulation abnormalities, hemolysis, inflammation, and clinical trajectory
An abnormal finding prompts further assessment	Interprets the overall pattern in relation to mechanism, clinical context, and trajectory to determine whether surveillance, reassessment, or targeted intervention is warranted

The IHN approach is a conceptual framework for integrating heterogeneous laboratory, functional, clinical, and circuit information. It is not a validated diagnostic or treatment algorithm.

**Table 4:** Developmental and Clinical Modifiers Relevant to Anticoagulation During Pediatric ECMO.

Domain	Developmental or Clinical Consideration	Potential Relevance to Anticoagulation
Endogenous anticoagulant pathways	Antithrombin and proteins C and S exhibit age-dependent concentrations and maturation trajectories, with particularly low levels during the neonatal period [25,26].	Developmental differences may modify endogenous regulation of coagulation and the response to anticoagulant therapy. Reduced antithrombin availability may attenuate unfractionated heparin activity, while anticoagulation measurements require interpretation in the context of developmental hemostasis and assay characteristics.
Coagulation factors	Procoagulant-factor concentrations vary during development [25,26].	Age-related baseline differences may affect conventional coagulation tests, particularly aPTT, and complicate interpretation of anticoagulant effect.
Antithrombin availability	UFH activity depends on antithrombin availability [12].	Antithrombin activity may warrant assessment when UFH responsiveness appears inadequate or anticoagulation measurements are discordant.
Body size and circuit-to-patient relationship	Body size, circuit surface area, priming volume, and the ratio of extracorporeal to circulating blood volume vary with patient size [29].	The relative magnitude of circuit exposure may differ across pediatric age and size groups and should inform interpretation of the clinical context.
Clinical indication and hemostatic state	ECMO indication, bleeding or thrombotic manifestations, recent surgery, organ function, and circuit conditions vary among patients [12,43].	Anticoagulation intensity should be individualized according to the evolving clinical state and competing bleeding and thrombotic risks rather than developmental stage alone.

**Table 5:** Clinical Contexts Prompting Reassessment of UFH Anticoagulation Intensity.

Clinical Context	Considerations Before Modifying UFH Intensity
Clinically significant bleeding	Reassess anticoagulant effect and monitoring-assay validity, including antithrombin availability when relevant to UFH activity. Evaluate circuit flow and stasis, cannula position, evidence of circuit dysfunction or thrombus progression, hemolysis, and ongoing coagulation or platelet activation. Integrate these findings to identify potentially correctable contributors before adjusting anticoagulation intensity.
Stable clinical course	Maintain the institutionally established anticoagulation strategy and monitoring plan, with serial integration of laboratory, clinical, and circuit findings and reassessment if the clinical trajectory changes.
Progressive patient or circuit thrombosis	Reassess anticoagulant effect and monitoring-assay validity, including antithrombin availability when relevant to UFH activity. Evaluate circuit flow and stasis, cannula position, evidence of circuit dysfunction or thrombus progression, hemolysis, and ongoing coagulation or platelet activation. Integrate these findings before considering changes in anticoagulation intensity.
Discordant anticoagulation assays	Assess potential biological, analytical, and preanalytical causes of discordance. Repeat or confirm testing when appropriate and interpret results in the context of the patient’s clinical condition, circuit findings, and concurrent hemostatic abnormalities. Determine which assay, or combination of assays, best informs UFH activity in the specific clinical context before adjusting anticoagulation intensity.
Mixed bleeding and thrombotic manifestations	Characterize the severity and trajectory of both bleeding and thrombotic manifestations and assess their potentially coexisting or interacting mechanisms. Reassess anticoagulant effect and the broader hemostatic profile, including the bleeding source, flow and stasis, cannula position, circuit pathology, hemolysis, developmental and systemic contributors, and recent surgery or procedures. Prioritize clinically consequential, potentially reversible contributors and individualize management according to the dominant mechanisms and immediate clinical risks, rather than assuming that an intermediate anticoagulation target will adequately address both processes.

This table provides a conceptual framework for interpreting clinical contexts that may influence UFH management. It does not define state-specific therapeutic targets or constitute a validated pediatric ECMO anticoagulation algorithm.

**Table 6:** Targeted Blood-Component and Hemostatic Therapy During Pediatric ECMO.

Intervention	Clinical Context	Key Considerations
Platelet transfusion	Clinically significant bleeding; postcardiotomy ECMO during the periprocedural period; or a high-bleeding-risk procedure, particularly when thrombocytopenia or clinically relevant platelet dysfunction is present.	In clinically relevant bleeding, the PEACE consensus suggests considering a platelet count of at least > 100 × 10^9^ cells/L; a higher threshold may be considered when platelet dysfunction is suspected. Similar targets may be considered during the periprocedural period after cardiopulmonary bypass or for high-bleeding-risk procedures. These recommendations are based on limited pediatric evidence and should be individualized. Consider platelet count and trajectory, functional platelet information and VET-derived clot strength when available, fibrinogen status, developmental stage, anticoagulant effect, and thrombotic risk. In nonbleeding patients outside these specific contexts, no universally validated pediatric ECMO transfusion threshold has been established.
Plasma	Clinically significant bleeding with suspected or demonstrated deficiency of multiple coagulation factors.	The PEACE consensus considers an initial plasma transfusion reasonable when INR is greater than 1.5 in a patient with clinically relevant bleeding. Interpret INR in the context of bleeding severity and trajectory, developmental hemostasis, anticoagulant effect, and the likely cause of prolongation. Consider fibrinogen and VET findings when available. Repeated plasma transfusions solely to correct INR may not improve outcomes; avoid transfusion solely to normalize an isolated laboratory abnormality.
Fibrinogen concentrate/cryoprecipitate	Clinically significant bleeding associated with reduced fibrinogen concentration or impaired functional fibrin contribution; postcardiotomy ECMO during the periprocedural period; or a high-bleeding-risk procedure.	For patients after cardiopulmonary bypass and those undergoing high-bleeding-risk procedures, the PEACE consensus suggests considering fibrinogen levels above 150 mg/dL during the periprocedural period. In periprocedural bleeding, this target may be maintained until bleeding ceases or decreases to minimal or moderate severity. These are weak recommendations based on limited pediatric evidence, not a universal threshold for all ECMO patients. Fibrinogen concentrate provides a more standardized dose; cryoprecipitate provides variable fibrinogen together with additional hemostatic proteins, including factor VIII and von Willebrand factor. Individualize according to bleeding severity and trajectory, procedural risk, and available functional assessment.
RBC transfusion	Clinically significant anemia, active bleeding, or inadequate oxygen delivery	Restrictive strategies using a threshold around 7 g/dL have been supported in selected stable critically ill children, although pediatric ECMO patients were not represented in the major randomized trials.
PCC	Exceptional rescue therapy for refractory, life-threatening bleeding	Consider only after source control and conventional multimodal therapy. Provides concentrated coagulation factors and may increase thrombotic risk, which is particularly relevant during ECMO.
rFVIIa	Exceptional rescue therapy for refractory, life-threatening bleeding	Consider only after source control and conventional multimodal therapy. Potent thrombin generation may promote patient and circuit thrombosis; use should therefore be highly restricted.
Antifibrinolytic therapy	Clinically significant bleeding or anticipated high risk of bleeding, particularly in surgical or high-risk procedural settings	Tranexamic acid or ε-aminocaproic acid may be considered as part of multimodality blood management. Evidence or clinical suspicion of excessive fibrinolysis may provide additional support. Potential thrombotic risk should be considered.

**Table 7:** Circuit-Based Strategies for Hemostatic Optimization During Pediatric ECMO.

Circuit Strategy	Primary Mechanism Addressed	Potential Hemostatic Implication
Biocompatible circuit surfaces and coatings	Reduce blood–surface interactions, protein adsorption, and contact activation	May reduce some components of platelet, coagulation, complement, and inflammatory activation; the magnitude of clinical benefit remains uncertain
Minimize unnecessary blood–biomaterial interface	Reduce artificial-surface exposure	May limit coagulation, platelet, and inflammatory activation
Reduce priming volume when feasible	Limit hemodilution and unnecessary artificial-surface exposure	May help preserve circulating hemostatic substrate concentrations and potentially reduce transfusion requirements
Optimize cannula size and position	Improve drainage and reduce abnormal flow and shear	May reduce flow instability, mechanical blood trauma, and hemolysis
Optimize pump speed and blood flow	Balance adequate support and drainage against excessive shear and stasis	May limit shear-mediated blood injury while maintaining effective circuit function
Prevent recurrent suction or chattering events	Reduce excessive negative pressures and flow instability	May reduce hemolysis and mechanical blood trauma
Avoid unnecessary circuit manipulation	Reduce additional blood–biomaterial and air exposure	May limit additional coagulation, complement, and inflammatory activation
Avoid unnecessary circuit or oxygenator exchanges	Avoid renewed blood–biomaterial exposure and procedural burden	May reduce additional thromboinflammatory activation when exchange is not clinically necessary
Promptly investigate progressive fibrin deposition or circuit thrombosis	Identify evolving circuit pathology and its mechanical and hemostatic consequences	May facilitate recognition of increasing thrombotic burden, hemolysis, or oxygenator dysfunction and inform subsequent circuit management
Circuit or oxygenator intervention when clinically consequential	Remove or replace a component compromising circuit function or patient support	May reduce ongoing circuit-related thrombogenicity and mechanical blood injury when the circuit has become a clinically significant source of dyshemostasis

These strategies are presented as mechanistic and clinical considerations rather than as validated interventions with established effects on pediatric ECMO outcomes.

**Table 8:** Evidence Base and Limitations of Hemostatic Management Strategies in Pediatric ECMO.

Clinical Domain	Evidence Source	Study Design and Population	Main Findings Relevant to Pediatric ECMO	Limitations and Implications
Bleeding and overall hemostatic complications	Challenges in Maintaining the Hemostatic Balance in Children Undergoing ECMO	Systematic review of pediatric ECMO studies	Identified substantial heterogeneity in hemostatic complications, risk factors, anticoagulation practices, and clinical outcomes.	Predominance of observational studies and inconsistent outcome definitions limit comparisons and generalizability.
	Bleeding Definitions in Pediatric ECMO Studies	Systematic review and meta-analysis	Identified substantial variability in bleeding definitions and reported bleeding prevalence across pediatric ECMO studies.	Inconsistent definitions and outcome ascertainment limit comparisons across cohorts and interpretation of pooled estimates.
Hemostatic monitoring	Coagulation in Pediatric ECMO: A Systematic Review of Studies Shows Lack of Standardized Reporting	Systematic review of pediatric ECMO studies	Identified variability in coagulation monitoring, reporting practices, and transfusion thresholds.	Inconsistent reporting and outcome definitions limit comparisons between studies and the establishment of universal targets.
	PEACE: Anticoagulation Monitoring and Targets	Systematic-review-informed consensus	Addresses interpretation and use of ACT, aPTT, anti-Xa, and complementary monitoring approaches.	Available pediatric evidence does not establish a single optimal monitoring strategy or universally applicable therapeutic targets.
Hemostatic Factor Replacement, Blood Component Transfusion, and Adjunctive Therapies	PEACE: Monitoring and Replacement of Antithrombin, Fibrinogen, and von Willebrand Factor	Systematic-review-informed consensus	Addresses laboratory assessment and replacement of selected hemostatic components during pediatric ECMO.	Evidence remains insufficient to define universally applicable replacement thresholds or support routine antithrombin supplementation.
	PEACE: Prophylactic Transfusion Strategies in Children Supported by Extracorporeal Membrane Oxygenation	Systematic-review-informed consensus	Provides a framework for platelet and other blood-component transfusion decisions in the context of bleeding risk and clinical circumstances.	Consensus recommendations informed by limited pediatric evidence.
	PEACE: Antifibrinolytic and Adjunct Hemostatic Agents	Systematic-re view-informed consensus	Evaluates antifibrinolytics and adjunctive therapies, including prothrombin complex concentrate and recombinant activated factor VII.	Limited pediatric evidence and potential thrombotic complications support selective, mechanism-directed use.
	ECSTATIC	Multicenter randomized feasibility trial involving 50 children receiving ECMO	Evaluated the feasibility of comparing platelet transfusion thresholds of 5 and 9 × 10^9^/L.	Designed to assess feasibility rather than establish efficacy, safety, or superiority of either threshold.
Anticoagulation: bivalirudin versus unfractionated heparin	McMichael	Pilot randomized controlled trial in neonatal and pediatric ECMO	Directly compared bivalirudin with unfractionated heparin in pediatric ECMO.	Pilot design and limited sample size preclude definitive conclusions regarding comparative efficacy and safety.
	Liu et al.	Systematic review and meta-analysis; 12 studies, 770 neonatal and pediatric patients, including one RCT and 11 observational studies	Bivalirudin was associated with lower odds of major bleeding and systemic thrombosis. No significant differences were found in circuit thrombosis or time within the therapeutic range.	Observational evidence predominated, limiting causal interpretation. The findings suggest potential benefit but do not establish superiority over unfractionated heparin.
Antithrombin supplementation	Elliott et al.	Systematic review and meta-analysis; 11 studies, including one RCT and 10 observational studies; seven studies involved pediatric populations	No significant overall differences were found in bleeding, thrombosis, or mortality. The pediatric subgroup showed an association between antithrombin supplementation and increased thrombotic events	Heterogeneity and predominantly observational evidence limit causal interpretation. The pediatric finding represents a potential safety signal rather than definitive evidence of harm.

## Data Availability

No new data were created or analyzed in this study. Data sharing is not applicable to this article.

## Abbreviations

ACT	activated clotting time
aPTT	activated partial thromboplastin time
AT	antithrombin
CPB	cardiopulmonary bypass
CRRT	continuous renal replacement therapy
dTT	dilute thrombin time
DTI	direct thrombin inhibitor
ECLS	extracorporeal life support
ECMO	extracorporeal membrane oxygenation
ELSO	Extracorporeal Life Support Organization
FIBTEM	fibrin-based thromboelastometry
HEPTEM	heparinase-modified thromboelastometry
IHN	Integrated Hemostatic Network
INR	international normalized ratio
PAI-1	plasminogen activator inhibitor-1
PCC	prothrombin complex concentrate
PEACE	Pediatric ECMO Anticoagulation CollaborativE
PT	prothrombin time
RBC	red blood cell
RCT	randomized controlled trial
rFVIIa	recombinant activated factor VII
ROTEM	rotational thromboelastometry
TEG	thromboelastography
t-PA	tissue plasminogen activator
TT	thrombin time
UFH	unfractionated heparin
VAD	ventricular assist device
VET	viscoelastic testing
vWF	von Willebrand factor
VV-ECMO	veno-venous extracorporeal membrane oxygenation
